# Tailored biochar from oil palm trunk via controlled carbonization for efficient dye adsorption

**DOI:** 10.1186/s40643-026-01013-8

**Published:** 2026-02-11

**Authors:** Mohd Idham Hakimi, Mohammed Abdillah Ahmad Farid, Mohd Nor Faiz Norrrahim, Mohd Rafein Zakaria, Yoshihito Shirai, Mohd Ali Hassan, Mohd Zulkhairi Mohd Yusoff

**Affiliations:** 1 Department of Bioprocess Technology, Faculty of Biotechnology and Biomolecular Sciences, Universiti Putra Malaysia (UPM), 43400 Serdang, Selangor Malaysia; 2 Laboratory of Biopolymer and Derivatives, Institute of Tropical Forestry and Forest Products (INTROP), Universiti Putra Malaysia (UPM), 43400 Serdang, Selangor Malaysia; 3 Research Centre for Chemical Defence (CHEMDEF), Universiti Pertahanan Nasional Malaysia, Kem Perdana Sungai Besi, 57000 Kuala Lumpur, Malaysia; 4 Laboratory of Processing and Product Development, Institute of Plantation Studies, Universiti Putra Malaysia (UPM), 43400 Serdang, Selangor Malaysia; 5 Department of Biological Functions Engineering, Graduate School of Life Science and Systems Engineering, Kyushu Institute of Technology, 2-4 Hibikino, Wakamatsu-ku, Kitakyushu, 808-0196 Japan

**Keywords:** Oil palm trunk, Biochar, Carbonization, Bioadsorbent, Methylene blue adsorption, Lignocellulosic biomass

## Abstract

Oil palm trunks (OPT) represent an underutilized agricultural byproduct that poses significant environmental challenges. The effectiveness of OPT as feedstock for biochar production will be depends on carbonization conditions, yet the relationship between process parameters and biochar properties remains insufficiently explored. This study investigates the potential of converting OPT into micropores bioadsorbent through controlled carbonization. Biochar was produced at temperatures of 300, 400, and 500 °C, with residence times of 2, 3, and 4 h, and subsequently characterized for its physicochemical properties and adsorption capacity. The results indicate that biochar produced at 300 °C for 2 h exhibited the highest surface area (10.24 m^2^/g), while the carbon content peaked at 79.9% in biochar synthesized at 500 °C for 4 h. Notably, although the maximum surface area was observed at 300 °C for 2 h, superior MB removal (52.5%) at longer residence time (4 h) indicates that adsorption performance was governed primarily by surface functional chemistry and pore accessibility rather than surface area alone. The enhanced adsorption at mild carbonization was attributed to the preservation of oxygen-containing surface functional groups rather than surface area alone. Langmuir isotherm analysis provided the best fit (R^2^ > 0.9), yielding a maximum monolayer adsorption capacity of 3.57 mg g^−1^ and a favourable separation factor (R_L_ < 1). These results demonstrate that adsorption performance of OPT-derived biochar is governed by surface chemistry controlled through carbonization severity, positioning OPT as a promising low-cost precursor for sustainable dye adsorption applications.

**Graphical abstract:**

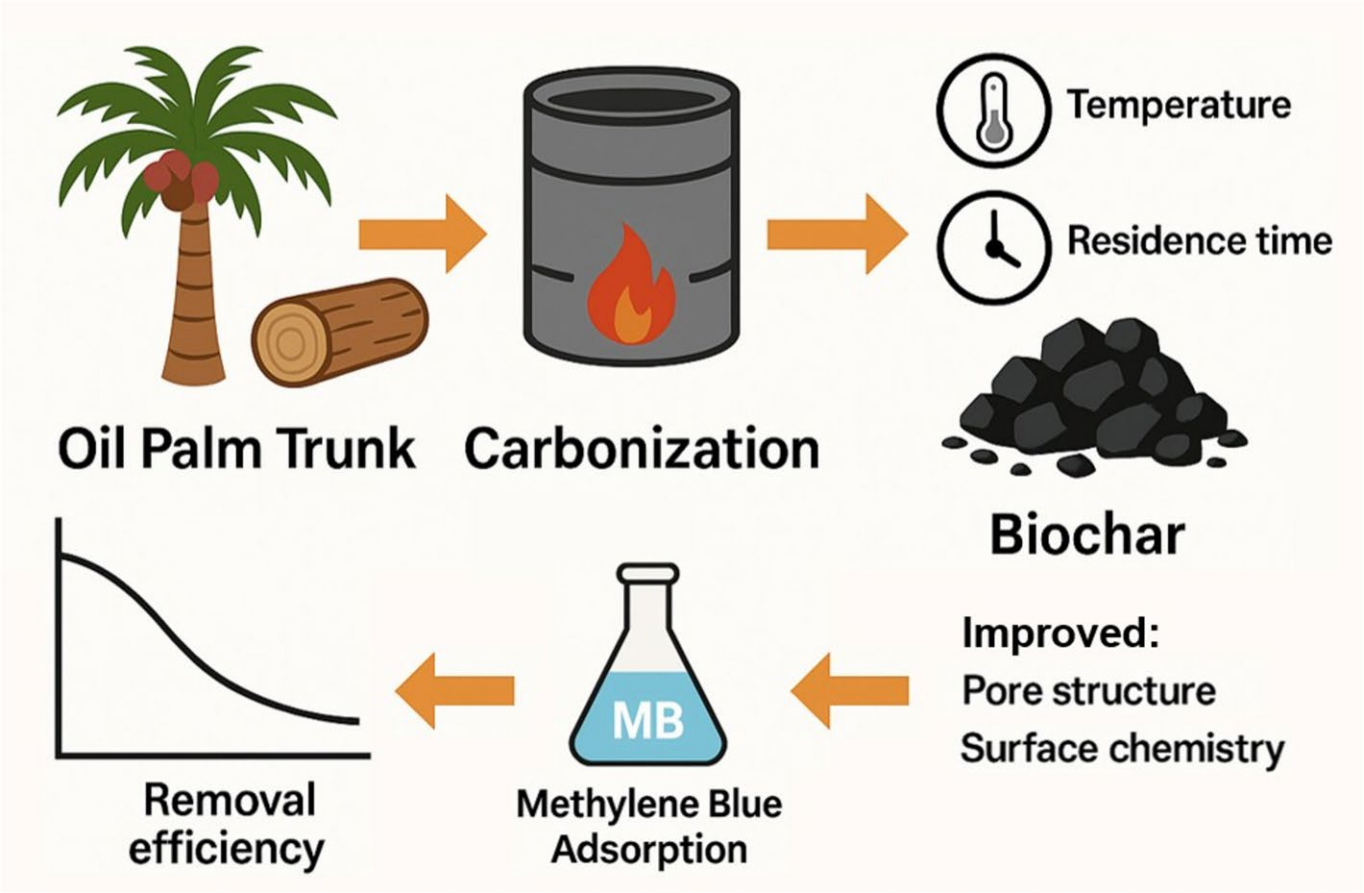

## Introduction

The global palm oil industry plays a pivotal role in economic growth, particularly in Malaysia, where approximately 5.65 million hectares of land are cultivated for oil palm production, generating over 21.8 million tonnes of biomass annually (Liu et al. 2025; Katibi et al. 2024a). Among the major agricultural residues, oil palm trunks (OPT) contribute nearly 14.9% of the total biomass, equating to an estimated 21.8 million tonnes per year (Katibi et al. 2021; Silva Medeiros et al. 2025). Despite their abundance, OPT remains largely underutilized, often left to decay or incinerated, leading to significant environmental concerns, including greenhouse gas emissions associated with unmanaged biomass decomposition (Katibi et al. 2021, 2024b). Given the urgency of sustainable biomass management, the transformation of OPT into high-value products such as biochar presents an innovative solution to mitigate waste while contributing to environmental remediation.

Biochar, a carbon-rich material produced via thermochemical processes such as pyrolysis, has gained increasing attention as an effective adsorbent for water purification due to its tunable surface area, hierarchical porosity, and functionalized surface chemistry (Lu et al. 2024; Yang et al. 2025). Compared with conventional activated carbon, biochar offers a more sustainable and cost-effective alternative by valorizing abundant waste biomass. Although biochars derived from oil palm residues, including empty fruit bunches (EFB), palm kernel shells (PKS), and oil palm fronds (OPF), have been extensively studied, recent comparative analyses emphasize that adsorption performance depends strongly on both feedstock chemistry and activation strategy, particularly when benchmarking non-activated versus activated biochars (Katibi et al. 2024b; Shitu et al. 2025). Within this context, oil palm trunk (OPT) remains markedly underexplored despite constituting one of the largest lignocellulosic biomass streams generated during oil palm replanting cycles.

Importantly, OPT differs fundamentally from other oil palm residues in both anatomical structure and chemical composition. Unlike the fibrous morphology of EFB or the shell-dominated structure of PKS, OPT exhibits a woody vascular framework with abundant parenchymatous tissue, lower inorganic ash content, and a lignin–cellulose distribution more akin to hardwood biomass. These characteristics govern distinct thermal decomposition pathways during carbonization, influencing devolatilization behaviour, pore development, and surface functionalization. Recent studies comparing lignocellulosic biochars derived from agricultural residues demonstrate that such feedstock-specific attributes critically determine adsorption behaviour, particularly in non-activated systems (Yang et al. 2025; Shitu et al. 2025). Consequently, the adsorption performance of OPT-derived biochar cannot be directly extrapolated from other oil palm residues. Nevertheless, existing studies on OPT biochar remain fragmented and largely focus on activated carbon production or fuel-related properties, with limited mechanistic insight into adsorption-relevant structure–function relationships.

In this study, the effects of carbonization temperature and residence time on the physicochemical properties and adsorption performance of OPT-derived biochar were systematically investigated. Carbonization temperatures of 300, 400, and 500 °C were selected to capture key stages of lignocellulosic thermal transformation, including volatile release, aromatization, and pore development (Lu et al. 2024; Katibi et al. 2024b). Lower temperatures favour the retention of oxygen-containing functional groups that promote hydrogen bonding, whereas higher temperatures enhance aromaticity and π–π interactions, thereby influencing dye adsorption behaviour (Yang et al. 2025; Shitu et al. 2025). Residence times of 2–4 h were employed to examine time-dependent structural evolution while maintaining practical energy efficiency and acceptable yields (Katibi et al. 2024b).

Methylene blue (MB) was employed as a model cationic dye due to its sensitivity to surface chemistry and porosity. Recent benchmarking studies on MB adsorption highlight that activated and engineered biochars generally exhibit higher adsorption capacities; however, these improvements arise primarily from post-processing rather than intrinsic feedstock behaviour (Lu et al. 2024; Yang et al. 2025). In contrast, non-activated biochars produced under controlled thermal conditions can still achieve effective MB removal through preserved oxygen-containing functionalities (Katibi et al. 2024b; Shitu et al. 2025). Within this framework, OPT-derived biochar represents a relevant model system for disentangling carbonization-driven effects from activation-induced enhancements.

Beyond adsorption performance, the utilization of OPT aligns with broader circular economy strategies for agro-industrial biomass valorization. Recent sustainability-oriented studies emphasize that redirecting underutilized agricultural residues into functional carbon materials supports waste reduction, resource efficiency, and closed material loops within biomass-intensive value chains (Katibi et al. 2024b; Shitu et al. 2025). Accordingly, this study demonstrates that OPT can be effectively transformed into a value-added bioadsorbent, supporting national sustainability and circular economy objectives (Liu et al. 2025; Katibi et al. 2024b).

## Materials and methods

### Materials

Oil palm trunks (OPT) were sourced from Felda Keratong, Pahang, Malaysia. The freshly harvested trunks were cut into smaller sections and air-dried for 7–10 days to reduce their initial moisture content, which typically ranges between 55 and 70%. Subsequently, the dried OPT was further processed into chips of approximately 1.5–2 cm^3^ using a mechanical grinder to enhance surface area and ensure uniformity. To prevent microbial degradation and ensure consistency in subsequent thermal processing, the OPT chips were oven-dried at 105 °C for 24 h until the moisture content was reduced to below 10%.

### Biochar synthesis

Oil palm trunk (OPT) biomass was carbonized under limited-oxygen conditions using covered crucibles placed in a muffle furnace (Carbolite Gero, UK). Dried OPT particles (< 2 mm) were weighed (10.0 ± 0.1 g per batch) into alumina crucibles (internal diameter: 50 mm; height: 40 mm; volume ≈ 75 mL) and sealed with tight-fitting alumina lids to restrict oxygen ingress during thermal treatment (Zhou et al. 2024). The crucibles were positioned in the furnace hot zone with consistent spacing to ensure uniform heat transfer. Carbonization was conducted by heating from ambient temperature to the target setpoints of 300, 400, or 500 °C, followed by isothermal holding for 2, 3, or 4 h, respectively. Carbonization at 300 °C favours initial devolatilization with retention of oxygen-containing functional groups, while 400 °C enhances aromaticity through cellulose and hemicellulose degradation. The 500 °C condition corresponds to higher-severity treatment dominated by lignin decomposition and structural stabilization. Residence times of 2, 3, and 4 h were employed to evaluate time-dependent property evolution while avoiding excessive thermal exposure. A constant heating rate of 10 °C min^−1^ was used to ensure uniform thermal decomposition. After carbonization, samples were cooled naturally in the furnace to minimize oxidation, then ground and sieved to < 150 µm for subsequent characterization and adsorption experiments. Volatile gases generated during carbonization were vented through the furnace exhaust, and no condensable products were recovered. As the muffle furnace is not a sealed flow reactor and no inert purge gas was employed, complete exclusion of oxygen cannot be guaranteed. Accordingly, the process is described as limited-oxygen carbonization rather than fully inert pyrolysis. This clarification is important, as oxygen ingress may influence biochar yield and surface chemistry, particularly under mild carbonization conditions.

### Biochar characterization

#### Proximate and ultimate analysis

Proximate analysis was conducted using a thermogravimetric analyser (EXSTAR6000, Hitachi, Japan) to determine moisture content, volatile matter, fixed carbon, and ash content following ASTM D1762-84 standards. Ultimate analysis, assessing the elemental composition (C, H, N, S, and O), was performed using a CHNS analyser (Vario EL III, Elementar, Germany). Oxygen content was determined by difference, considering the total elemental composition.

#### Structural analysis

Structural analysis was performed using Fourier Transform Infrared (FTIR) spectroscopy (PerkinElmer Spectrum GX, USA) over the spectral range of 4000–450 cm^−1^ with a resolution of 4 cm^−1^. The identification and assignment of functional groups were carried out to elucidate the chemical characteristics of the biochar surface before and after the adsorption process, as FTIR provides insight into surface functionalities responsible for pollutant binding, such as hydroxyl, carboxyl, and aromatic groups (Katibi et al. 2021; Hakimi et al. 2024; Hassan et al. 2021; Tomczyk et al. 2020).

#### Surface area and porosity

The Brunauer–Emmett–Teller (BET) surface area, total pore volume, and pore size distribution of the biochar samples were determined using nitrogen adsorption–desorption analysis with a Micromeritics TriStar II Plus instrument (USA). Prior to analysis, the samples were degassed at 150 °C for 8 h under vacuum to eliminate adsorbed moisture and volatile organics. The Barrett-Joyner-Halenda (BJH) method was employed to evaluate mesopore size distribution, while micropore volume was estimated using the t-plot method. These analyses are essential for characterizing the porous architecture of biochar, which directly influences its adsorption capacity and is known to vary significantly with pyrolysis conditions (Katibi et al. 2021; Hassan et al. 2021; Tomczyk et al. 2020; Zubair et al. 2020).

### Adsorption studies

#### Methylene blue adsorption assay

The adsorption performance of OPT biochar was evaluated using methylene blue (MB) as a model organic pollutant. Batch adsorption experiments were conducted by mixing a fixed amount of biochar (1 g L^−1^) with 100 mL of MB solution at an initial concentration of 25 mg L^−1^. The mixture was continuously agitated at 150 rpm in a temperature-controlled orbital shaker (25 °C) for 120 min to ensure equilibrium adsorption. To assess the influence of initial MB concentration, additional experiments were conducted at 10, 50, and 100 mg L^−1^, while the effect of solution pH was examined by adjusting the pH between 3 and 10 using 0.1 M HCl or NaOH solutions. Ionic strength was not independently controlled, and no background electrolyte was added, allowing adsorption behaviour to be evaluated under simplified aqueous conditions. As the point of zero charge (pHₚzc) of the biochar was not measured, electrostatic interactions are discussed based on established literature rather than direct surface charge determination.

#### Determination of adsorption capacity

The residual MB concentration was measured using a UV–Vis spectrophotometer (Thermo Scientific Genesys 30) at an absorbance wavelength of 663 nm. The adsorption capacity (q_e_) and removal efficiency were calculated using the following Eq. 1.

1 $$ q_{e} = \frac{{\left( {C_{o} - C_{e} } \right)V}}{m} $$

where C_o_ and C_e_ are the initial and equilibrium concentrations of MB (mg/L), is the volume of the solution (L), and is the mass of biochar used (g). All experiments were conducted in triplicate to ensure data reproducibility, and results were expressed as mean ± standard deviation.

#### Adsorption isotherm study

To evaluate the adsorption behavior of conditioned biochar towards methylene blue (MB), batch adsorption experiments were conducted. A fixed dosage of biochar (10 g L^−1^) was added to 25 mL Erlenmeyer flasks containing MB solutions at varying initial concentrations (20, 40, 60, 80, and 100 mg L^−1^). The pH of each solution was adjusted to 7 prior to the experiment to minimize pH-related variability. The flasks were sealed and agitated at 150 rpm for 24 h at an ambient temperature of 30 °C to ensure equilibrium. The adsorption data were analysed using three isotherm models, which is Langmuir (Eq. 2), Freundlich (Eq. 3), and Temkin (Eq. 4), to understand the adsorption mechanism and to determine the best-fit model. Adsorption isotherm and kinetic parameters were estimated using non-linear regression to minimize error distortion associated with linearized model forms.

2 $$ \frac{{{\mathrm{C}}_{{\mathrm{e}}} }}{{{\mathrm{q}}_{{\mathrm{e}}} }} = \frac{{{\mathrm{C}}_{{\mathrm{e}}} }}{{{\mathrm{q}}_{{\mathrm{m}}} }} + \frac{1}{{\left( {{\mathrm{K}}_{{\mathrm{L}}} {\mathrm{q}}_{{\mathrm{m}}} } \right)}} $$

where, q_e_ is the adsorption capacity at equilibrium (mg g^−1^) and C_e_ is the equilibrium concentration of the adsorbed species (mg mL^−1^). Langmuir constant and maximum adsorption capacity, denoted as K_L_ (L mg^−1^) and q_m_ (mg g^−1^).

3 $$ {\mathrm{Q}}_{{\mathrm{e}}} = {\mathrm{K}}_{{\mathrm{F}}} + {\mathrm{C}}_{{\mathrm{e}}}^{{1/{\mathrm{n}}}} $$

where, qe denotes the quantity of adsorbate adsorbed per unit mass of adsorbent (mg g^−1^) and C_e_ signifies the equilibrium concentration of adsorbate in solution (mg/mL). Denoted as K_F_ (mg g^−1^) (L mg^−1^)^1/n^ and n, the Freundlich constant and adsorption intensity can be determined by examining the relationship between log q_e_ and log C_e_ on a graph.

4 $$ {\mathrm{q}}_{{\mathrm{e}}} = \frac{{{\mathrm{RT}}}}{{\mathrm{b}}}\ln {\mathrm{K}}_{{\mathrm{T}}} + \frac{{{\mathrm{RT}}}}{{\mathrm{b}}}\ln {\mathrm{C}}_{{\mathrm{e}}} $$

where, K_T_ represents the equilibrium bonding constant (L mol^−1^) associated with the highest bonding energy, b denotes the heat of adsorption, R signifies the universal gas constant (8.314 J K^−1^ mol^−1^), and T designates the temperature in Kelvin (K).

## Results and discussion

### Effect of carbonization parameters on biochar yield

The yield of biochar is a critical determinant in assessing the economic feasibility and scalability of biochar production for industrial applications. In this study, biochar yield exhibited an inverse relationship with carbonization temperature, consistent with thermal decomposition principles. At 300 °C, biochar yield was recorded at 34.3%, whereas at 500 °C, yield decreased to 29.3%, reflecting a 14.6% reduction (Table 1). This decline can be attributed to the progressive breakdown of hemicellulose (200–300 °C), cellulose (300–400 °C), and lignin (400–600 °C), which results in increased volatilization of organic compounds, leaving behind a denser carbonaceous structure. Previous studies on biomass pyrolysis have reported similar trends, with biochar yields typically ranging between 25 and 40%, depending on feedstock composition and thermal parameters (Liu et al. 2025; Katibi et al. 2024a).

**Table 1:** Biochar yield at different carbonization conditions

Biomass	Resident time (h)	Temperature (°C)	Label	Yield (wt%)	Surface area (m^2^/g)	Total pore volume (cm^3^/g)	Average pore diameter (nm)	References
Empty fruit bunch	2.00	550	EFBBC	27.7	1.53	0.11	282.5	Nalaya et al. 2020)
Palm kernel shell	1.50	500	PKSBC	–	238.00	0.29	–	Kong et al. 2019)
Oil palm frond	0.75	550	OPFBC	26.0	0.34	–	160.6	Shrivastava et al. 2021)
Oil palm trunk	0.75	550	OPTBC	27.0	0.36	–	142.8	Shrivastava et al. 2021)
	2.00	300	OPTBC-2-300	34.3 ± 1.1	10.24	0.03	2.0	This study
		400	OPTBC-2-400	31.6 ± 0.8	9.80	0.03	2.0	
		500	OPTBC-2-500	30.7 ± 0.7	3.45	0.01	3.2	
	3.00	300	OPTBC-3-300	33.2 ± 0.8	1.70	0.03	3.4	
		400	OPTBC-3-400	31.8 ± 0.9	1.22	0.01	3.6	
		500	OPTBC-3-500	29.4 ± 1.5	1.63	0.01	2.8	
	4.00	300	OPTBC-4-300	33.1 ± 0.3	1.05	0.01	3.4	
		400	OPTBC-4-400	31.2 ± 0.6	2.34	0.01	3.0	
		500	OPTBC-4-500	29.3 ± 0.4	1.51	0.01	3.2	

Residence time also influenced yield, albeit to a lesser extent compared to temperature. At 300 °C, increasing the residence time from 2 to 4 h led to a marginal reduction in yield from 34.3 to 33.1%, whereas at 500 °C, the decline was more pronounced, from 30.7 to 29.3% (Table 1). This observation suggests that extended thermal exposure enhances the release of volatile organic compounds but has a diminishing effect at lower temperatures. The reduction in yield with prolonged carbonization time is consistent with studies on lignocellulosic biomass, which suggest that extended residence times promote further devolatilization and enhance fixed carbon content (Katibi et al. 2021; Silva Medeiros et al. 2025).

Beyond the quantitative decline in yield, carbonization temperature and residence time significantly affected biochar composition and thermal stability. Higher carbonization temperatures result in biochars with greater fixed carbon content and lower hydrogen-to-carbon (H/C) and oxygen-to-carbon (O/C) ratios, which are indicative of increased aromaticity and structural stability. A study by Hakimi et al. (Lu et al. 2024) reported that biochars produced at temperatures above 500 °C exhibit an H/C ratio below 0.3, indicative of a highly stable, graphitic-like structure. This shift is particularly relevant for adsorption applications, where high-carbon-content biochars exhibit superior chemical resistance and enhanced pollutant binding capacity (Yang et al. 2025).

**Fig. 1:**
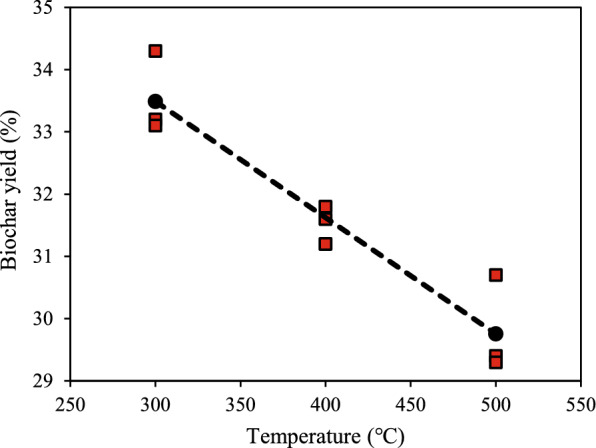
Effect of carbonization temperature on biochar yield

As illustrated in Fig. 1, biochar yield consistently decreases with increasing carbonization temperature across all residence times. This trend aligns with established pyrolysis mechanisms, wherein the progressive thermal degradation of biomass components leads to a reduction in solid carbonaceous residues. The figure further highlights that while lower temperatures retain more biochar mass, higher temperatures facilitate the production of biochars with improved stability and functionality, crucial for advanced environmental applications.

Comparing these findings with other lignocellulosic biochars, OPT-derived biochar demonstrates yield values comparable to oil palm frond biochar (27–35%) and EFB biochar (26–33%) but slightly lower than palm kernel shell biochar, which can reach up to 40% due to its higher lignin content (Katibi et al. 2024b; Shitu et al. 2025). Studies on coconut shell biochar indicate even lower yield values (18–27%) due to its dense structural composition and high volatile matter content (Zhou et al. 2024). These comparisons highlight the importance of feedstock selection and process optimization to achieve desired material properties while maintaining economic viability.

### Physicochemical properties of OPT biochar

#### Proximate and ultimate analysis

The proximate and ultimate analyses provide essential insights into the composition and stability of biochar, which directly influence its applications in adsorption and soil amendment. Proximate analysis indicated a significant reduction in volatile matter content as the carbonization temperature increased. Biochar produced at 300 °C exhibited a volatile matter content of 20.1%, whereas at 500 °C, it was reduced to 5.5% (Table 2). This reduction is attributed to the progressive degradation of hemicellulose, cellulose, and lignin during pyrolysis, which leads to the release of volatile organic compounds and the formation of a more thermally stable carbon matrix. Similar reductions in volatile matter with increasing carbonization temperatures have been observed in other biomass studies, with values ranging between 5 and 15% at temperatures exceeding 500 °C (Katibi et al. 2021; Hassan et al. 2021).

**Table 2:** Proximate and ultimate analysis of OPT biochar

Biochar	Proximate analysis (%)				Ultimate analysis (%)					Atomic ratio		
	Moisture content	Volatile matter	Fixed carbon	Ash	C	H	N	S	O	H/C	O/C	(O + N)/C
OPT Raw (Hakimi et al. 2024)	NA	82.3	9.7	8.0	40.5	6.2	1.0	0.07	51.1	0.20	1.30	1.30
OPTBC-2-300	2.3	20.1	59.2	18.4	63.8	4.1	1.2	0.15	30.7	0.06	0.48	0.50
OPTBC-2-400	2.0	9.4	78.5	10.1	72.1	3.3	1.2	0.11	23.3	0.05	0.32	0.34
OPTBC-2-500	1.9	7.4	82.3	8.4	76.7	2.7	0.8	0.11	19.8	0.03	0.26	0.27
OPTBC-3-300	2.2	17.0	66.1	14.7	64.7	4.0	1.0	0.07	30.2	0.06	0.47	0.48
OPTBC-3-400	1.4	8.1	81.8	8.7	70.4	3.3	1.4	0.09	24.9	0.05	0.35	0.37
OPTBC-3-500	1.3	6.6	83.8	8.3	78.7	2.5	1.0	0.11	17.7	0.03	0.23	0.24
OPTBC-4-300	1.8	11.3	77.9	9.0	66.6	3.9	0.9	0.04	28.6	0.06	0.43	0.44
OPTBC-4-400	1.1	6.4	83.9	8.6	73.7	3.3	0.8	0.05	22.2	0.04	0.30	0.31
OPTBC-4-500	1.0	5.5	87.6	6.0	79.9	2.6	0.9	0.08	16.5	0.03	0.21	0.22

Ultimate analysis showed an increase in carbon content from 63.8% at 300 °C to 79.9% at 500 °C, accompanied by a decline in oxygen content. Atomic ratios (H/C, O/C, and (O + N)/C) were calculated on a molar basis from ultimate analysis data following standard practice. The hydrogen-to-carbon (H/C) ratio decreased below 0.3 at higher temperatures, indicating a shift toward a more aromatic and graphitic structure (Table 2). This transformation is critical for biochar stability and its resistance to microbial degradation. Studies have reported that biochars with H/C ratios below 0.3 exhibit enhanced structural integrity and longevity in environmental applications (Tomczyk et al. 2020).

**Fig. 2:**
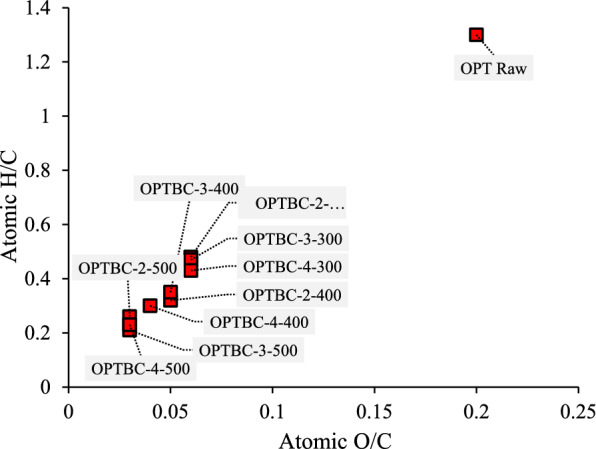
Van Krevelen diagram of OPT biochars

Atomic ratios (H/C and O/C) were calculated on a molar basis from ultimate analysis results. The low H/C values observed reflect extensive dehydrogenation of OPT biochar at elevated carbonization severity. The Van Krevelen diagram (Fig. 2) further illustrates the reduction in H/C and O/C atomic ratios with increasing carbonization temperature and residence time. This trend is attributed to dehydration and decarbonylation reactions, which lead to the release of hydroxyl-rich volatile compounds such as methanol, acetic acid, and water (Wyn et al. 2020), resulting in an increasingly aromatic and thermally stable biochar matrix.

#### Functional group analysis

**Fig. 3:**
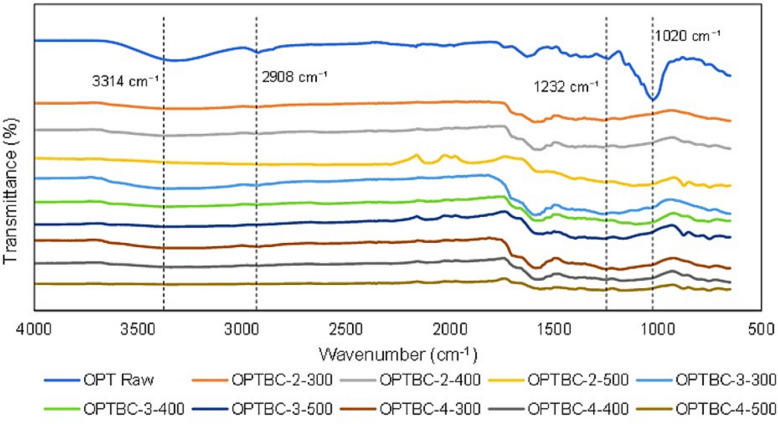
FTIR spectra of OPT biochar at different carbonization temperatures

Fourier transform infrared spectroscopy (FTIR) analysis was conducted to identify the chemical functionalities present in the biochar and their changes with increasing carbonization temperature. As illustrated in Fig. 3, biochar produced at 300 °C exhibited strong absorption peaks at 3314 cm^−1^, corresponding to hydroxyl (–OH) stretching, and 1725 cm^−1^, indicative of carbonyl (C=O) groups. These functional groups are associated with lignocellulosic biomass and play a crucial role in adsorption interactions (Hassan et al. 2021).

With increasing temperature, a significant reduction in hydroxyl and carboxyl functional groups was observed. At 500 °C, the intensity of these peaks diminished, while the presence of aromatic C=C bonds (1572 cm^−1^) became more prominent, indicating increased carbonization and aromaticity (Tomczyk et al. 2020). The loss of oxygen-containing functional groups at higher temperatures is consistent with biochars produced from empty fruit bunches and palm kernel shells, where carboxyl and hydroxyl groups decrease by approximately 60–80% between 300 and 500 °C (Lawal et al. 2020).

#### Surface area and porosity

Brunauer–Emmett–Teller (BET) analysis confirmed the strong influence of carbonization temperature on OPT biochar porosity. Biochar produced at 300 °C exhibited the highest surface area (10.24 m^2^ g^−1^), which decreased markedly to 3.45 m^2^ g^−1^ at 500 °C (Table 1). This decline is attributed to micropore shrinkage and structural collapse at elevated temperatures, a behaviour commonly reported for lignocellulosic biochars and other oil palm residues beyond 400 °C (Lawal et al. 2020).

These results highlight the trade-off between porosity retention and thermal severity during carbonization. Lower temperatures (300–400 °C) preserve surface area and oxygen-containing functional groups, favouring adsorption performance, whereas higher temperatures (> 500 °C) enhance structural stability and carbonization degree, which are more relevant for soil amendment and carbon sequestration. In this respect, OPT biochar exhibits surface characteristics comparable to oil palm frond and empty fruit bunch biochars, though with lower surface area than palm kernel shell biochar (> 200 m^2^ g^−1^), reflecting differences in feedstock lignin content (Salleh et al. 2019).

The relatively low BET surface areas observed for OPT biochars (≤ 10.24 m^2^ g^−1^) are characteristic of non-activated biochars produced under mild carbonization and do not indicate incomplete carbonization or pore blockage. In the absence of activation, pore development is governed primarily by devolatilization and matrix rearrangement rather than extensive micropore formation. This is supported by FTIR evidence of progressive volatile removal with increasing temperature and nitrogen adsorption behaviour indicative of limited intrinsic microporosity. Consequently, adsorption performance is controlled predominantly by surface functional chemistry and accessible pore domains rather than total surface area alone.

### Nitrogen adsorption isotherm and pore size distribution

**Fig. 4:**
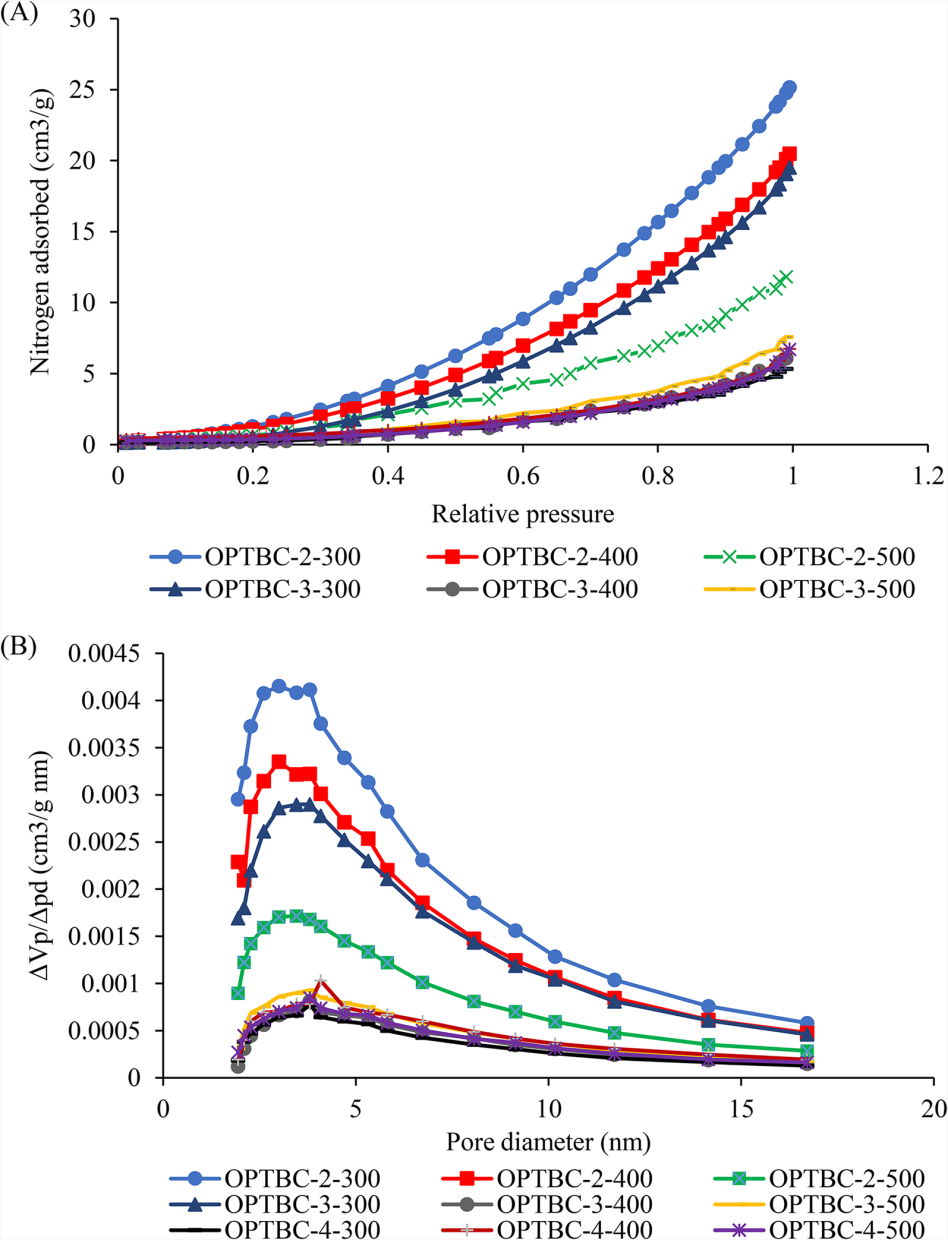
**A** Nitrogen adsorption–desorption isotherms and **B** pore size distribution of OPT biochars

The nitrogen adsorption–desorption isotherms and pore size distributions of OPT biochars are shown in Fig. 4. The isotherms do not strictly conform to a single ideal IUPAC type, but are characteristic of non-activated biochars with low surface area, exhibiting weak adsorbate–surface interactions and enhanced adsorption at higher relative pressures due to multilayer adsorption. Accordingly, the isotherms are discussed qualitatively rather than being assigned rigidly to a single IUPAC classification. The adsorption profiles show limited uptake at low relative pressure (P/P₀ < 0.1) followed by increased nitrogen adsorption at higher P/P₀, which is typical of materials dominated by external surface adsorption and larger accessible pores (Alothman 2012).

The presence of pores in the micropore range (< 2 nm, IUPAC definition) is suggested by the pore size distribution; however, their overall contribution is limited, consistent with the modest BET surface areas of the biochars. OPTBC-2-300 exhibited the highest nitrogen adsorption capacity, indicating comparatively greater pore accessibility under mild carbonization conditions. Increasing carbonization temperature and residence time resulted in reduced nitrogen uptake, reflecting structural densification and partial pore collapse under more severe thermal treatment.

OPTBC-2-400 displayed a representative pore size centred at approximately 1.7 nm, with a corresponding pore volume of 0.002 cm^3^ g^−1^ nm^−1^, suggesting that intermediate carbonization conditions may facilitate limited pore restructuring without extensive collapse (Lawal et al. 2020; Samsudin et al. 2022). Textural properties summarized in Table 1 show that biochar produced at 300 °C achieved the highest BET surface area (10.24 m^2^ g^−1^) and total pore volume (0.03 cm^3^ g^−1^), whereas biochar carbonized at 500 °C exhibited substantially lower surface area (3.45 m^2^ g^−1^) and pore volume (0.01 cm^3^ g^−1^), accompanied by an increase in geometrically estimated average pore diameter, indicative of fewer but broader accessible pores. These trends reflect the balance between pore development and collapse during pyrolysis, consistent with previous reports on lignocellulosic biochars (Hassan et al. 2021; Zhang et al. 2012).

Although OPT biochars exhibit relatively modest surface areas compared with activated carbons, the presence of limited microporosity (≈ 1–2 nm) supports their adsorption potential. Given that the molecular size of methylene blue (~ 1.43 nm) approaches this pore size range, adsorption is expected to be governed primarily by accessible pore entrances, external surfaces, and surface functional groups, rather than complete penetration into all micropore domains. Moreover, nitrogen adsorption conducted under cryogenic conditions does not fully represent diffusion behaviour in aqueous systems, reinforcing that adsorption performance is controlled mainly by surface chemistry and pore accessibility rather than total pore volume.

### Morphology of OPT biochar

**Fig. 5:**
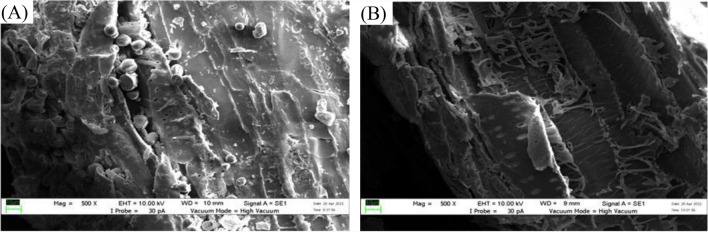
SEM morphology of **A** OPT raw and **B** OPT biochar

Figure 5 illustrates the SEM micrographs of raw and carbonized OPT, clearly demonstrating the morphological evolution induced by pyrolytic treatment. The surface of the raw OPT (Fig. 5A) is characterized by a dense, compact matrix with irregular topography and negligible porosity. Following carbonization (Fig. 5B), structural transformations are observed, including a smoother surface attributed to the release of volatile compounds, indicative of increased graphitic ordering (Farid et al. 2024). Concurrently, the appearance of rough, uneven, and cracked surface reflects the thermal degradation of lignocellulosic components and the development of a porous architecture, which is critical for adsorption application (Farid and Andou 2018). This is in agreement with (Islam et al. 2024), which reported that the presence of surface cracks and fissures on biochar contributes to its structural macroporosity, thereby facilitating enhanced diffusion of adsorbates into the internal pore network.

### Adsorption performance of OPT biochar

**Fig. 6:**
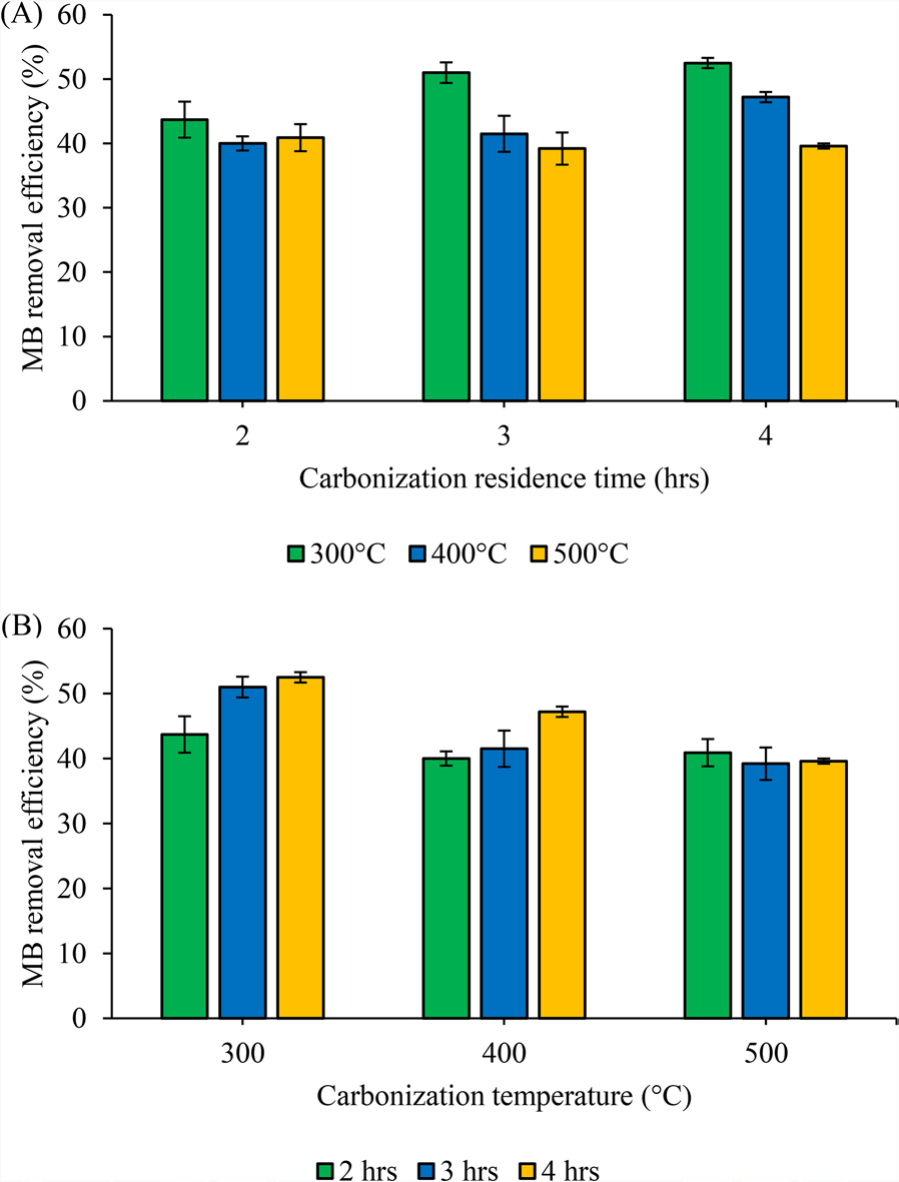
Effect of **A** residence time and **B** carbonization temperature on MB removal efficiency

The adsorption capacity of biochar is influenced by several interrelated physicochemical parameters, including surface area, pore volume, surface chemistry, and carbonization conditions. In this study, the adsorption performance of OPT biochar was evaluated using MB as a model organic pollutant. As summarized in Table 3 and illustrated in Fig. 6, the performance data highlight the influence of carbonization conditions on MB adsorption efficiency. The results revealed that biochar produced at 300 °C for 4 h exhibited the highest MB removal efficiency, reaching 52.5%, which corresponds to an adsorption capacity (q_e_) of 1.93 mg/g. This value is consistent with adsorption capacities reported for other lignocellulosic biochars without chemical activation, which typically range between 1 and 5 mg/g for MB removal (Hassan et al. 2021; Lawal et al. 2020).

**Table 3:** Comparative MB removal between OPT biochar and other oil palm residue biochars

Biochar sources	Carbonisation conditions		MB removal capacity (mg/g)	References
	Resident time (h)	Temperature (℃)		
EFB	0.50	450	8.70	Ibrahim et al. 2017)
OPMF	0.17	650	3.90	Aziz et al. 2022)
OPT	2.00	300	1.61	
		400	1.47	
		500	1.5	
	3.00	300	1.88	
		400	1.53	This study
		500	1.44	
	4.00	300	1.93	
		400	1.74	
		500	1.46

Despite its relatively low surface area (10.24 m^2^/g at 300 °C), the superior performance of OPT biochar at lower temperatures can be attributed to its abundance of oxygen-containing surface functional groups, such as hydroxyl and carboxyl moieties. According to previous studies, the mechanism of MB adsorption onto biochar is primarily dependent on the dispersive π-π interactions between the aromatic ring structure of MB and the graphene-like sheets of the biochar (Huff et al. 2014; Zubair et al. 2020). Therefore, these groups did enhance electrostatic interactions and hydrogen bonding with cationic dyes MB. As the carbonization temperature increased, a marked decline in MB removal efficiency was observed. At 500 °C, the removal efficiency dropped below 30%, indicating that excessive thermal treatment reduces the availability of active functional groups, even though it increases carbon content and aromaticity, as evidenced in both Fig. 6 and the declining trend shown in Table 3.

Similar findings have been reported in studies on palm kernel shell and bamboo biochars, where adsorption capacity decreased by over 40% when pyrolysis temperature increased from 350 to 600 °C due to the loss of functional groups (Yang et al. 2025; Salleh et al. 2019). Additionally, MB adsorption is generally favoured by higher pore accessibility and surface polarity rather than surface area alone. For example, biochar produced from coconut shell with surface areas above 200 m^2^/g showed lower MB uptake than biochar with active surface functional groups and moderate porosity (Katibi et al. 2021).

Figure 6A shows the effect of carbonization residence time on MB removal, revealing that an increase in residence time correlates with an increase in MB adsorption. This could be attributed to the greater availability of vacant active sites and functional groups on the biochar surface, which enhance adsorption capacity (Liu et al. 2025). Conversely, Fig. 6B highlights the impact of carbonization temperature on MB adsorption. The results indicate that as the carbonization temperature increases, MB adsorption decreases. For instance, biochars produced with a 4 h residence time showed the highest MB removal at 300 °C (52.5%) and the lowest at 500 °C (39.6%). This reduction in MB adsorption is likely due to a decrease in the availability of vacant active sites and active functional groups on the biochar surface as the carbonization temperature increases.

### Adsorption isotherm

Adsorption isotherm analysis was conducted to elucidate the underlying adsorption mechanism and surface interactions of OPT biochar with MB. Understanding isotherm behavior is essential for interpreting the adsorbent’s capacity, surface heterogeneity, and binding affinity. To this end, three widely accepted theoretical models, Langmuir, Freundlich, and Temkin isotherms, were applied to the experimental data.

**Fig. 7:**
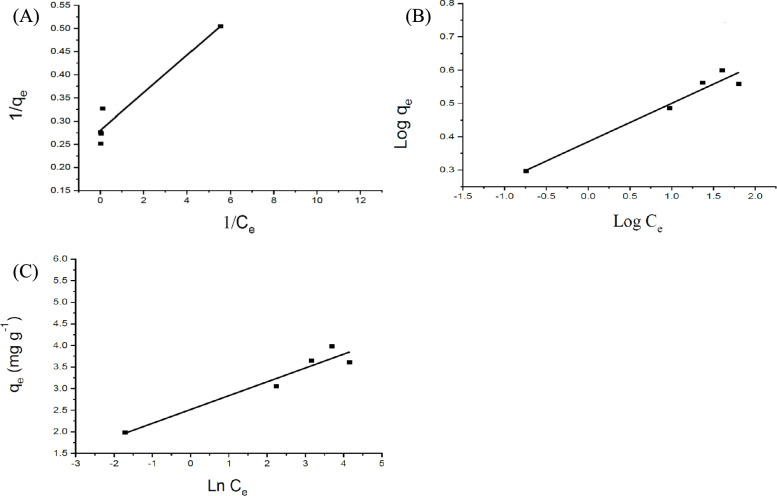
Fitted adsorption isotherm curves for OPT biochar using the **A** Langmuir, **B** Freundlich, and **C** Temkin models

As presented in Fig. 7 and Table 4, all three models demonstrated strong correlations with the OPT biochar data, each yielding R^2^ values above 0.9. The Langmuir constant (K_L_) represents the affinity between adsorbate and adsorption sites, whereas the maximum adsorption capacity (qmax) reflects the finite number of accessible sites. In the present study, the relatively high K_L_ values indicate strong interaction between MB and surface functional groups of OPT biochar, while the modest qmax values (≤ 1.93 mg g^−1^) are consistent with the low BET surface areas and limited pore accessibility of the non-activated materials. The Langmuir separation factor (R_L_) decreased from 0.014 to 0.0014 as the initial methylene blue concentration increased from 10 to 100 mg L^−1^, reflecting operation within a high-affinity adsorption regime while remaining within the favourable range (0 < R_L_ < 1).

**Table 4:** Equilibrium model parameters for adsorption of MB by OPT biochar

Isotherm model	Parameter	OPT biochar
Langmuir	q_max_ (mg g^−1^)	3.57
	K_L_ (L mg^−1^)	6.91
	RL at C_0_ = 10 mg L^−1^	0.014
	RL at C_0_ = 50 mg L^−1^	0.0029
	RL at C_0_ = 100 mg L^−1^	0.0014
	R^2^	0.91
Freundlich	K_f_	2.43
	1/n	0.12
	R^2^	0.94
Temkin	B_T_ (J mol^−1^)	0.32
	K_T_ (L mg^−1^)	2555.1
	R^2^	0.90

The Temkin model also showed excellent agreement, highlighting the influence of indirect interactions during the adsorption process. Importantly, the separation factor (RL) for OPT biochar was less than one (R_L_ = 0.0018), indicating highly favourable adsorption of MB within the investigated concentration range. Herein, the Langmuir model provided the best fit, implying monolayer adsorption behaviour with a calculated maximum adsorption capacity of 3.57 mg/g. It should be noted that a very low R_L_ value reflects strong adsorbent–adsorbate affinity under Langmuir assumptions and does not imply irreversible adsorption. As desorption or regeneration behaviour was not evaluated in this study, the adsorption process cannot be classified as irreversible based solely on the R_L_ parameter.

## Conclusion

Oil palm trunk (OPT) was evaluated as a non-activated biochar precursor for methylene blue (MB) adsorption under controlled carbonization conditions. Biochar yields ranged from 29.3–34.3 wt%, decreasing with increasing carbonization severity. Among the tested conditions, biochar produced at 300 °C for 4 h exhibited the highest MB removal efficiency (52.5%) with a maximum adsorption capacity of 1.93 mg g^−1^, despite a modest BET surface area (10.24 m^2^ g^−1^). Although the adsorption capacity is lower than that of activated carbons, the observed performance is consistent with other non-activated lignocellulosic biochars and is governed primarily by surface functional groups and pore accessibility rather than extensive microporosity. This performance is attributed to the preserved surface functionalities such as –OH and –COOH, governing adsorption through electrostatic and hydrogen bonding interactions. Increasing the carbonization temperature to 500 °C resulted in increased carbon content up to 79.9% and fixed carbon to 87.6%, while reducing H/C and O/C ratios to 0.03 and 0.21, indicating greater aromaticity. However, this structural transformation led to a decline in MB removal (39.6%) and surface area (3.45 m^2^/g), highlighting a trade-off between surface chemistry and stability. Furthermore, isotherm analysis using MB as the model adsorbate showed excellent fits (R^2^ > 0.9) for all three models. The Langmuir model best described the data, indicating monolayer adsorption with a maximum capacity of 3.57 mg/g, while the Temkin model suggested indirect interactions. A separation factor (R_L_ < 1) confirmed the process was favourable and efficient. Collectively, this study provides mechanistic insight into the process–structure–function relationships of OPT biochar and establishes OPT as a viable, low-cost, and sustainable bioadsorbent precursor. The results further suggest that moderate post-treatment activation could enhance adsorption performance while retaining the favourable surface chemistry, positioning OPT biochar as a promising material within circular biomass valorization and wastewater treatment frameworks.

## Data Availability

All data generated or analyzed during this study are included in this published article. Additional datasets used and/or analyzed during the current study are available from the corresponding author upon reasonable request.
